# Social determinants of health in rural Indian women & effects on intervention participation

**DOI:** 10.1186/s12889-023-15743-3

**Published:** 2023-05-19

**Authors:** Aarthi Arun, Manohar Prasad Prabhu

**Affiliations:** 1 Cornell University, Ithaca, NY 14850 USA; 2 Swami Vivekananda Youth Movement, Mysore, Karnataka India

**Keywords:** Social Determinants, Maternal health, Health Inequity, Pregnancy

## Abstract

The social determinants of health have become an increasingly crucial public health topic in recent years and refer to the non-medical factors that affect an individual’s health outcomes. Our study focuses on understanding the various social and personal determinants of health that most affect women’s wellbeing. We surveyed 229 rural Indian women through the deployment of trained community healthcare workers to understand their reasons for not participating in a public health intervention aimed to improve their maternal outcomes. We found that the most frequent reasons cited by the women were: lack of husband support (53.2%), lack of family support (27.9%), not having enough time (17.0%), and having a migratory lifestyle (14.8%). We also found association between the determinants: women who had lower education levels, were primigravida, younger, or lived in joint families were more likely to cite a lack of husband or family support. We determined through these results that a lack of social (both spousal and familial) support, time, and stable housing were the most pressing determinants of health preventing the women from maximizing their health outcomes. Future research should focus on possible programs to equalize the negative effects of these social determinants to improve the healthcare access of rural women.

## Introduction

### Social determinants of health

The World Health Organization defines the Social Determinants of Health (SDH or SDOH) as the conditions in which “people are born, grow, work, live, and age and the interactions of forces that shape the conditions of one’s daily life” [1]. Social determinants of health primarily fall into 3 categories as defined by the Commission on Social Determinants of Health under WHO. The first category is structural factors which refer to those that generate stratification and social class divisions in the society and are primarily influenced by the political and socioeconomic context of a given community. Examples of structural factors are Income, Education, Occupation, Social Class, Gender, Race/ethnicity. The second category is intermediary factors including broader factors such as material circumstances (such as housing and neighborhood quality), psychosocial circumstances (such as social support, and stressful living circumstance, behavioral and biological factors (such as nutrition and physical activity) [2].

In the past 20 years, various discussions and interventions focusing on the social determinants of health have emerged globally. Specifically, a large amount of research has focused on the importance of social determinants of health in early childhood to predict health outcomes in adulthood [3–5]. In addition, many studies have focused on the importance of SDOH to predict health outcomes in adulthood, especially among women. Prior research has found social support, socioeconomic position, ethnicity, and nature of their country’s health system were among the most essential factors in predicting women’s health outcomes [6, 7]. Social support is defined as, interpersonal transactions that involve emotional concern, instrumental aid, information, or appraisal [8]. However, the impact of social determinants on rural maternal health has not been as thoroughly researched.

This study focuses on the determinants that prevent women from participating in a health intervention program focused on improving their chances of a successful pregnancy and delivering a healthy child. These social and personal determinants can reveal the beliefs, characteristics, and thought processes that enable or limit women in making decisions related to their own health. Many social determinants for not participating in such healthy behaviors have been suggested including personal causes, such as concerns about privacy and time, and widely held beliefs, such as lack of trust in researchers and confusion about the goals of the study [9, 10].

From a statistical standpoint, low participation rates can lead to sampling bias when a significant number of people refuse to join a study. As a result, the sample may not accurately represent the desired population and can lead to non-response bias and a decrease in the statistical accuracy of the study.

### The initiative

Healthy Life Trajectories Initiative (HeLTI) is an international research collaboration between the Canadian Institutes of Health Research, the Department of Biotechnology (India), Medical Research Council (South Africa), and the National Natural Science Foundation (China), in collaboration with the World Health Organization. The study focuses on 4 linked cohorts that will assess the effects of interventions to lower the risk factors for noncommunicable disease (NCD) and promote early childhood development [11].

The Indian cohort is supervised by the Vivekananda Memorial Hospital (VMH) and focuses on rural women living in 2 sub-districts located near the Southern Indian city of Mysore: Heggadadevanakote (HD) Kote and Saragur. The intervention features 10 personalized educational modules that are delivered to local women by community health workers. The community health care workers are women from the aforementioned 2 sub-districts that are trained and paid by VMH to deploy modules directly to the women, and collect biospecimens and other clinical data (detailed below). The educational modules focused on maintaining a diverse diet, normal body weight, and an adequate intake of micronutrients before and during pregnancy, and the benefits of breastfeeding postnatally, and were delivered throughout the conception and pregnancy process. Depending on the group that the women were randomly assigned to, women will be delivered these modules prenatally or during pregnancy at monthly intervals. Women also participated in group parenting programs run by community health workers trained in cognitive behavioral therapy with the aim to encourage discussion to address perinatal depression and improve child development [11].

The study requires voluntary participation from the women who are expected to provide information about the health status and SDOH of both themselves and their families. This information will be collected by community health workers and will involve regular contact sessions by these health workers to disseminate the above-mentioned modules in the women’s homes.

It is envisioned that the results of this study will encourage women to participate in improving health outcomes and adopt clinically beneficial maternal health practices to support the development of both mothers and children. In order to monitor the improvement of health outcomes and practices, the community health workers also collect clinical data through biospecimens and data such as weight, height, and body composition at monthly intervals to monitor changes throughout the delivery of the educational modules [11].

## Methods

### Target population

Our study focused on rural Indian women aged 18–45 who have expressed interest in conceiving a child shortly. The women lived in the sub-districts of Heggadadevanakote (HD) Kote and Saragur located in the Indian state of Karnataka.

With regards to large scale statistics of both subdistricts, they have a combined population of 264,000 and a male-to-female sex ratio of 51:49. The female literacy rate is 60% as compared to the national average of 59.5% nationwide. In rural Karnataka, the total fertility rate is 1.8%. Specifically, young mothers are more prevalent in this area with 6.6% of women aged 15–18 pregnant, and 24.7% of women aged 20–24 being married before age 18 [12].

### Data collection

We used a cross-sectional study design that disseminated a survey to understand the beliefs and characteristics of our intended study population. This survey tool was designed to assess the perception of women and their willingness to participate in the study. The tool included questions that would help in identifying the most common reasons for non-participation among eligible women. In order to ensure validity of our data, we chose a cross sectional study with the goal of collecting data to better understand characteristics and beliefs of the women. The survey collected a variety of information on health determinants including social features such as ethnicity and family type (nuclear or joint), qne economic features such as annual income, employment status, and education level. Demographic information was also collected namely the number of years married, age, and the number of living children. A checklist was included with commonly cited reasons related to SDOH for not participating in interventions such as a migratory lifestyle, lack of spousal support, and lack of familial support [9, 10]. To ensure consistency in definition, we established “support” as the participant’s family or spouse agreement with or encouragement of her wish to take part in the study. Therefore, lack of spousal and familiar support encompass any situations in which the stated family member disagrees with the participant’s wish to engage in the study and thus causes them to deny their participation. In addition, since the study requires the participant’s time and thought, the participant’s families were also expected to provide support with regards to taking over some of their responsibilities and work during the study’s duration. Personal reasons were also listed including not enough time, not believing in the benefits of the intervention, prior negative experiences with hospital staff, and concerns about health privacy or risks of the program. Participants were able to pick as many of the above causes as they wished. A ranking system similar to the Likert Scale (1 for strongly disagree to 5 for strongly agree) was also used to gauge participants’ opinions on: the perceived benefits of the intervention, the intervention-hosting hospital, and researchers. We defined researchers as both the community health care workers and their supervisors at the hospital. These opinions were measured to understand if widespread mistrust in any of these groups or institutions could have been a possible barrier in participating in the wider intervention. We chose to incorporate a Likert scale as it is an efficient way to quantify beliefs, and opinions which traditionally cannot be precisely defined [13].

Eligible participants who did not wish to engage in the health interventions were identified by community health workers. The 229 participants were randomly selected using a simple random sample method from this group to take this survey. These community workers were trained at VMH according to global HelTI guidelines and received a standardized training module detailing how to properly deploy the survey. The survey was delivered to participants orally by the community health workers through the use of Kobo Humanitarian response software. In order to prevent variation across healthcare workers, participants were randomly assigned to community healthcare workers to survey. Community workers were instructed to verbalize each question in the survey and allow the participants to expand on certain points if they wished to. The survey also featured a short-answer question space for community workers to note down any points that the participant verbalized in their elaboration that was not included as a discrete question in the survey.

Associations between patient characteristics and reasons for opting out were analyzed using chi-square analysis through R statistical software. A significance level of 0.5 and a confidence level of 95% were used in all statistical analyses (Version 4.1.2).

## Results

### Personal & social characteristics

229 responses were collected over 5 days across 74 villages in the 2 sub-districts. Demographically, the majority of women were between the ages of 19–25, had been married between 2 and 5 years, and had been pregnant before. With regards to social characteristics, the majority of women had at minimum a high school degree or a college degree, were currently housewives, lived in a joint family, and had an estimated annual income of under 10,000 rupees 1 (< 126 USD) or an income between 10,000 and 20,000 rupees (126 to 252 USD).

### Descriptions of results

**Fig. 1:**
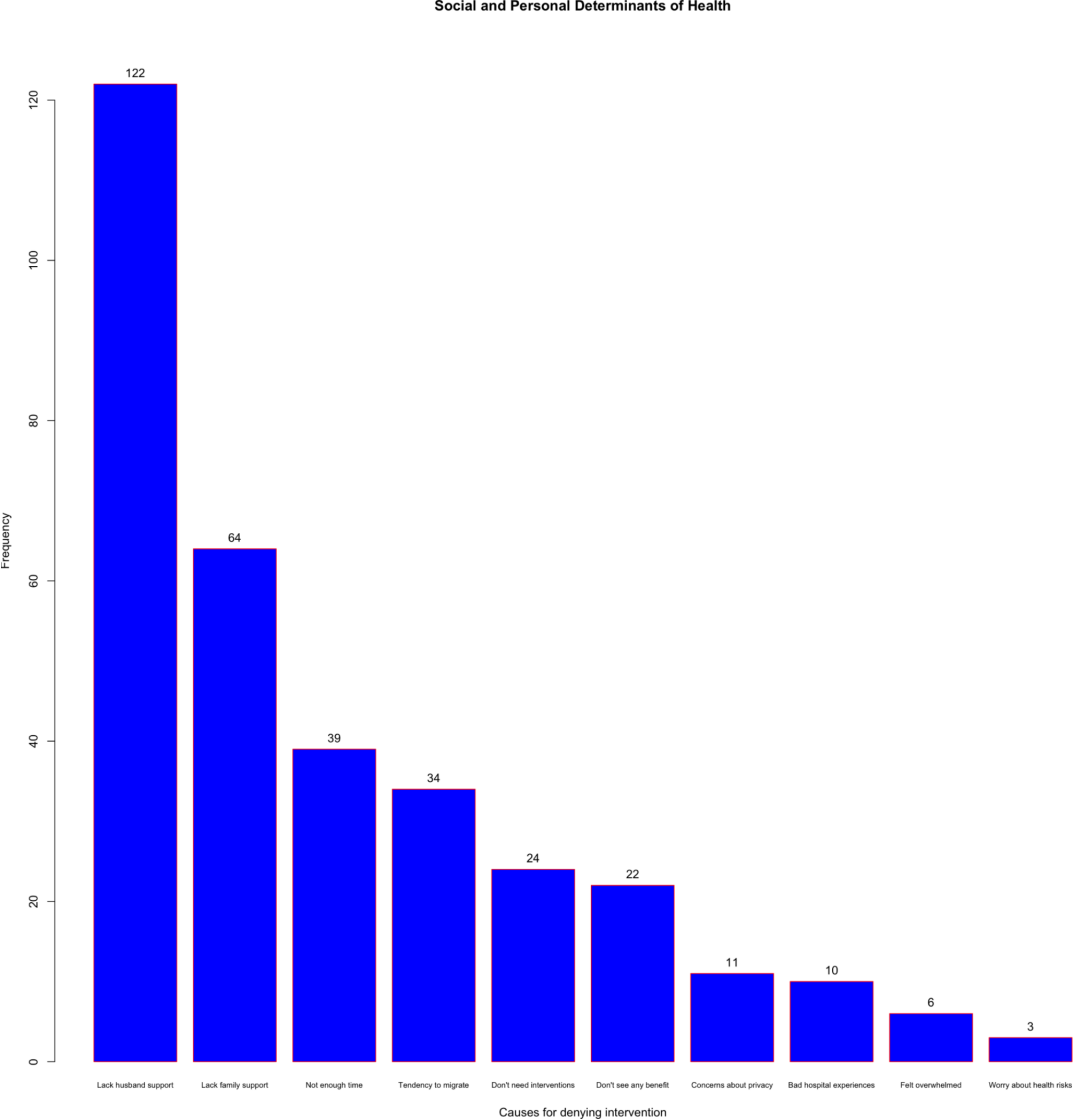
The various causes selected by women for denying participation in the intervention

Social and personal reasons for not engaging in the health intervention were summarized into 10 primary causes and their distribution is shown in Fig. 1. The reasons that the women cited were that they:

- Did not have enough time.
- Have a tendency to migrate or do not live in one place full time.
- Do not need the health interventions offered by the program.
- Had bad experiences with medical facilities or staff in the past.
- Did not see any clear benefits from the interventions.
- Had concerns about the privacy of health and personal data.
- Worry about health risks or side effects of program.
- Felt confused or overwhelmed by the study.
- Lacked support from their husband.
- Lacked support from their family.

The most common reasons for opting out of the health interventions were a lack of spousal support (53.2%), a lack of familial support (27.9%), not having enough time to participate (17.0%), and a tendency to migrate (14.8%).

**Fig. 2:**
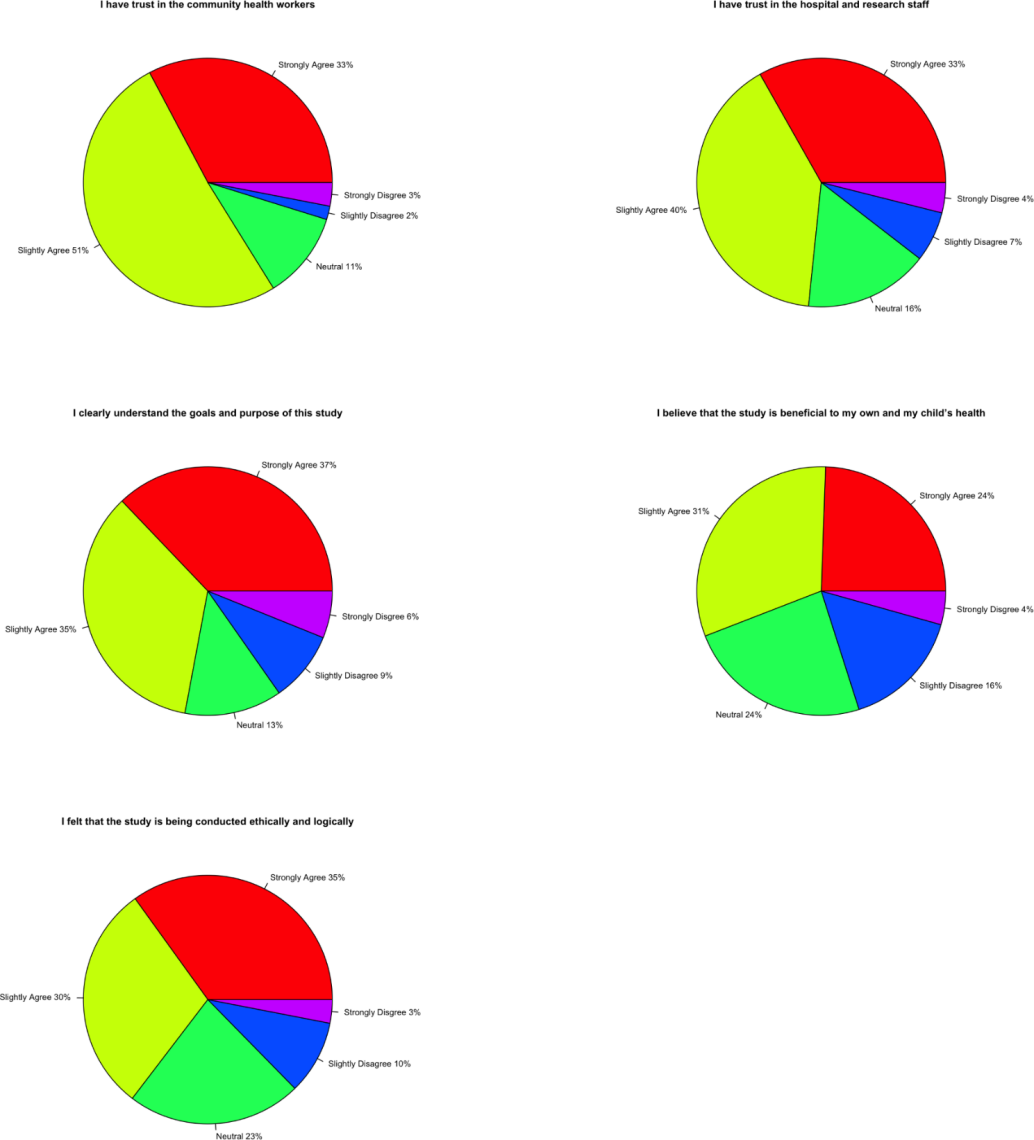
The women’s opinions on the study, staff, and the methods used in the project

With regards to the women’s opinions on the study, the majority of women agreed with all statements below as shown in Fig. 2:

- I have trust in the community health workers.
- I have trust in the research staff.
- I clearly understand the goals and purpose of this study.
- I believe that the study is beneficial to my own and my child’s health.
- I felt supported by family to participate in this project.
- I felt that the study is being conducted ethically and logically.

Chi-squared analyses between participant’s characteristics and causes for not pursuing interventions showed significant associations between multiple factors. One such association was between the highest level of a participant’s educational attainment and citing a lack of husband support: women with post-graduate level degrees were less likely to cite a lack of husband support. On the contrary, women whose highest education level was lower primary education or less were more likely to cite lack of husband support as a reason for not pursuing the intervention (p = 0.002). Family type and lack of family support were found to be linked as women who lived in nuclear families (husband, wife, and children) were less likely to cite a lack of family support as compared to women who lived in joint families (p = 0.0009). Age was also found to play a role in spousal support as younger women, aged between 18 and 24, were more likely to cite a lack of husband support (p = 0.04463) than older women. A relation was found between women who had not been pregnant before and to receiving family support, as women who had not previously carried were less likely to receive family support to participate in the intervention (p = 0.02).

## Discussion

Summarizing the results, the most common reason for women opting out of health-improving interventions was a lack of support from others, both from their husbands and their families. Support systems are a crucial component of the largest social determinant of health known as social cohesion and have been linked with greater physical and mental health outcomes. Support systems have been found to improve access to health-enhancing resources [14], increase health-related behaviors, and lessen the effect of psychological stressors [15]. The relationship is particularly important between socioeconomic resources and strong social support as women who did not receive adequate support from their families were unable to participate in interventions that would both improve their health and the health of their children.

This study also provides evidence for associations between various social and personal determinants of health. Lower education levels, larger family size, lower age, and women who were not previously pregnant were all classifications that were more likely to cite a lack of spousal or familial support. Women who fit into one or more of these categories are more likely to hold lesser sway regarding familial and personal decision-making with younger and less educated women generally having less decision-making power as they are seen by their male counterparts as “less capable” of handling this power [8]. This decision- making power held by women is further diluted in larger families as this responsibility is largely monopolized by the male heads of the family. As a result, many women may not be able to maximize their health outcomes as they do not possess the autonomy required to make their own health decisions [16]. Globally, a multitude of studies focusing on women has reported familial support to be a positively correlated predictor of health outcomes [17] with a large portion of these studies emphasizing partner support as particularly significant [18, 19]. On a larger scale, participation in the intervention also required the participants to forgo a small amount of time at home to engage with community workers. Families and spouses who discourage potential participants from this engagement likely display greater inequality in division of workload and caring for family members within the household as they are not able to cover these responsibilities during this time. This unfair division of household responsibilities is especially problematic in our target population of rural women, as patriarchal values with an emphasis on the childbearing and household responsibilities of women are especially prominent within this population [20]. These findings are exacerbated when considering women with lower education levels, who cited lower levels of spousal support in the study), also demonstrate more uneven distribution of household responsibilities than their more educated counterparts [21]. As a result, women in rural societies are more likely to suffer less life satisfaction and psychological wellbeing and greater rates of depression as these measures are directly correlated to low levels of spousal support [22].

Other than familial support, a lack of time was the third most cited reason for not participating in the health interventions. This finding supports similar studies that cite busy lifestyles as participants’ primary reason for not participating in health-improving programs [23]. Many have postulated that time itself was considered a social determinant of health as recent research has found that time-poor people have greater barriers to physical activity and poorer physical and mental health [24]. The consideration of time as a SDOH has become especially important in the last decade with increased labor participation and people spending more time working than before [25]. Studies have found that people who spend more time working are more likely to make poor health choices such as less physical activity [24] and prioritize their wellbeing less leading to poorer physical and mental health outcomes due to increased stress. This phenomenon of not having enough time for adequate health choices is known as “time poverty” and has also been found to affect women more. This gender inequality can be tied to the lack of female independence in rural households as time poverty is generally associated with less time autonomy [26]. This association arises as women whose time division is controlled by other people (spouses and families) are more likely to be overloaded with responsibilities and tasks.

In addition, the fourth most cited reason was a migratory lifestyle. A migratory lifestyle can be linked to the social determinant of health known as housing stability (which falls under the category of economic stability) and has been linked to poorer physical health and decreased access to health care [27–29]. The action of migration itself can lead to poorer health outcomes due to taxing travel conditions, and limited health access during migration [30]. In addition, the temporary living situations of migrants results in an inability to pursue long-term health interventions and programs that can improve their overall health. In addition, migrant women often face intersectional discrimination due to their identities as both migrants and women. This discrimination can be institutional and limit their access to adequate health care services leading to especially poor mental and physical health for migrant women [31]. Easing this discrimination with mobile clinics and culturally aware healthcare workers have been suggested to help address this inequality.

Both these findings reveal that the health interventions or lack of concern about their health were not preventing the women from improving their health outcomes but rather a limitation of various social factors. This conclusion was furthered by the fact that the majority of women agreed that the study and its staff were ethical and trustworthy. The presence of community healthcare workers likely contributed to this belief and strengthened the validity of the study.

We were able to effectively analyze a rural population by delivering the survey through a group of local community health workers. These workers came from the communities that the study itself was focusing on and thus had a greater understanding of the local context and population. This proved useful as women who had chosen to opt-out of the health interventions were still willing to work with the familiar community health workers to fill out the social determinant screening. In addition, the presence of community workers generally increased response rates as participants were more likely to favor direct oral communication as opposed to a written survey given both social relations and the proportion of illiterate women in the community. However, the study had some limitations in regards to its sample size because the study itself aimed to study women who opted out of the original intervention and subsequently were more likely to deny their participation in our additional studies.

Our study is particularly significant as it explores a previously understudied population and can be applied to a variety of disciplines. Though prior research has been performed on the social determinants of health among a variety of populations including both women, adolescents, and the elderly, rural women have not been studied on an international scale extensively before this study. Our results can also be applied to the field of research methodology by allowing researchers to understand the key features that encourage people to opt out of research studies. On a global scale, this research can be used to address the most pressing social determinants of health affecting rural women to improve the health outcomes of both themselves and their children.

## Conclusion

This study aims to find the key social determinants of health relevant to improving the outcomes of rural Indian women through a maternal health intervention. Our study found that spousal support, followed by familial support, was the most relevant social determinant of health in Indian rural women. Time and lack of stable housing were also found as impactful secondary determinants in the study. Future programs should also target the populations we found that were limited concerning social determinants including women with less education, younger women, primigravida women, and women living in joint families. In addition, future research should focus on ways to address these pressing determinants to allow greater healthcare access for women globally by targeting the social determinants of health in rural women to improve maternal health outcomes and improving the participation rates of women in health programs and longitudinal research studies.

## Data Availability

Datasets collected and analyzed during this study are not publicly available due to requirements related to protecting participant privacy but are available from the corresponding author on reasonable request.

## Abbreviations

SDOH/ SDH: Social determinants of Health
VMH: Vivekananda Memorial Hospital
HeLTI: Healthy Life Trajectories Initiative
NCD: Non-communicable disease
